# Creating a Modified Version of the Cambridge Multimorbidity Score to Predict Mortality in People Older Than 16 Years: Model Development and Validation

**DOI:** 10.2196/56042

**Published:** 2024-08-26

**Authors:** Debasish Kar, Kathryn S Taylor, Mark Joy, Sudhir Venkatesan, Wilhelmine Meeraus, Sylvia Taylor, Sneha N Anand, Filipa Ferreira, Gavin Jamie, Xuejuan Fan, Simon de Lusignan

**Affiliations:** 1 Nuffield Department of Primary Care Health Sciences University of Oxford Oxford United Kingdom; 2 Peninsula Medical School University of Plymouth Plymouth United Kingdom; 3 Medical & Payer Evidence Statistics, BioPharmaceuticals Medical AstraZeneca PLC Cambridge United Kingdom; 4 Medical Evidence, Vaccines and Immune Therapies AstraZeneca PLC Cambridge United Kingdom; 5 Royal College of General Practitioners of the United Kingdom London United Kingdom

**Keywords:** pandemics, COVID-19, multimorbidity, prevalence, predictive model, discrimination, calibration, systematized nomenclature of medicine, computerized medical records, systems

## Abstract

**Background:**

No single multimorbidity measure is validated for use in NHS (National Health Service) England’s General Practice Extraction Service Data for Pandemic Planning and Research (GDPPR), the nationwide primary care data set created for COVID-19 pandemic research. The Cambridge Multimorbidity Score (CMMS) is a validated tool for predicting mortality risk, with 37 conditions defined by Read Codes. The GDPPR uses the more internationally used Systematized Nomenclature of Medicine clinical terms (SNOMED CT). We previously developed a modified version of the CMMS using SNOMED CT, but the number of terms for the GDPPR data set is limited making it impossible to use this version.

**Objective:**

We aimed to develop and validate a modified version of CMMS using the clinical terms available for the GDPPR.

**Methods:**

We used pseudonymized data from the Oxford-Royal College of General Practitioners Research and Surveillance Centre (RSC), which has an extensive SNOMED CT list. From the 37 conditions in the original CMMS model, we selected conditions either with (1) high prevalence ratio (≥85%), calculated as the prevalence in the RSC data set but using the GDPPR set of SNOMED CT codes, divided by the prevalence included in the RSC SNOMED CT codes or (2) conditions with lower prevalence ratios but with high predictive value. The resulting set of conditions was included in Cox proportional hazard models to determine the 1-year mortality risk in a development data set (n=500,000) and construct a new CMMS model, following the methods for the original CMMS study, with variable reduction and parsimony, achieved by backward elimination and the Akaike information stopping criterion. Model validation involved obtaining 1-year mortality estimates for a synchronous data set (n=250,000) and 1-year and 5-year mortality estimates for an asynchronous data set (n=250,000). We compared the performance with that of the original CMMS and the modified CMMS that we previously developed using RSC data.

**Results:**

The initial model contained 22 conditions and our final model included 17 conditions. The conditions overlapped with those of the modified CMMS using the more extensive SNOMED CT list. For 1-year mortality, discrimination was high in both the derivation and validation data sets (Harrell C=0.92) and 5-year mortality was slightly lower (Harrell C=0.90). Calibration was reasonable following an adjustment for overfitting. The performance was similar to that of both the original and previous modified CMMS models.

**Conclusions:**

The new modified version of the CMMS can be used on the GDPPR, a nationwide primary care data set of 54 million people, to enable adjustment for multimorbidity in predicting mortality in people in real-world vaccine effectiveness, pandemic planning, and other research studies. It requires 17 variables to produce a comparable performance with our previous modification of CMMS to enable it to be used in routine data using SNOMED CT.

## Introduction

People with multimorbidity, defined by those with 2 or more long-term conditions (LTCs) [1-6], have complex needs and impose increasing demands on primary care services given the aging population. Multimorbidity is associated with reduced life expectancy [7], lower quality of life [8], and an increased risk of hospitalization and death due to COVID-19 [9]. In clinical trials, vaccination against COVID-19 showed reduced risk of hospitalization and death in all groups [10,11]. However, in real-world studies, people with multimorbidity benefited less from vaccination [12] and were at increased risk of mortality, morbidity, and hospitalization, compared to those without multimorbidity [13]. People with 5 or more LTCs had a more than 4-fold higher risk of severe COVID-19 outcomes than those with less than 5 LTCs [12].

Developing a single comorbidity measure is challenging [14]. The Charlson Comorbidity Index (CCI) is a commonly used tool to predict mortality over time [15]. However, CCI is based on hospital data, therefore, its applicability to primary care data is limited and not readily implementable [16]. The Cambridge Multimorbidity Score (CMMS) addressed this limitation and is an established measure of multimorbidity in primary care data. The original CMMS used 37 LTCs from routine primary care data in computerized medical records to predict the risk of primary care consultations, unplanned hospital admissions, and mortality [17]. It was developed and validated using the Clinical Practice Research Datalink [18] from the codes of Read version 2. However, Read version 2 is no longer used in England and is not updated since 2018 [19]. Additionally, the original CMMS model excluded people younger than 21 years of age, which somewhat restricted its applicability to the general population.

To overcome these limitations, we have already developed and validated a modified CMMS, replacing Read version 2 with the Systematized Nomenclature of Medicine clinical terms (SNOMED CT) [20] and using pseudonymized data from the Oxford Royal College of General Practitioners (RCGP) Research and Surveillance Centre (RSC) sentinel network of individuals aged 16 years or older [21]. Established in 1967, the RSC is an internationally renowned source of primary care data [22]. It has been used for influenza and respiratory disease monitoring for the last 50 years [23]. During the COVID-19 pandemic, with linkage to existing NHS (National Health service) England data sets, RSC data were also used to understand its epidemiology and assess vaccine effectiveness and safety [24-27]. This modified version of the CMMS was used to assess the real-world effectiveness of the Oxford-AstraZeneca COVID-19 vaccine in England (RAVEN) study, which was run on the RSC using linked data from NHS England [28].

The RAVEN study also used primary care data from the larger and nationwide General Practice Extraction Service Data for Pandemic Planning and Research (GDPPR) data source maintained by NHS England, providing pseudonymized data for over 54 million people in England [29]. GDPPR is linked at the individual patient level to hospital, death, vaccine exposure, and test results. However, while substantial, its primary care data collection is incomplete. The primary care data were created from the existing list of conditions comprising 56,319 different SNOMED CT codes. This is a large number, but it is less than 20% of all SNOMED CT codes. These covered some clinical conditions well (eg, diabetes) and some less so (eg, psychoactive substance disorder). This study aimed to develop and validate a modified version of the CMMS, which could be used for the population aged 16 years and older in this new English NHS nationwide data set (GDPPR).

## Methods

### UK Primary Care Data

In the United Kingdom, each patient registers with a single general practitioner practice. Information about their primary care consultations, prescriptions, investigation results, and certified sickness and mortality data are recorded in computerized medical records systems. Each patient has a unique identifier, the NHS number, which allows data linkage with other data sets, including the hospital data, Hospital Episode Statistics, death certificate data provided by the Office for National Statistics and the NHS prescribing data set [30].

### Data Sources

We used pseudonymized data from the RSC to construct and validate a revised version of CMMS based on the limited set of SNOMED CT codes (we refer to this as the GDPPR-modified CMMS). RSC data are stored in the Oxford RCGP Digital Informatics Hub (ORCHID) trusted research environment. The RCGP RSC extracts data from just under 2000 general practices in England [31] and provides a data set that is representative of individuals in England.

**Figure 1:**
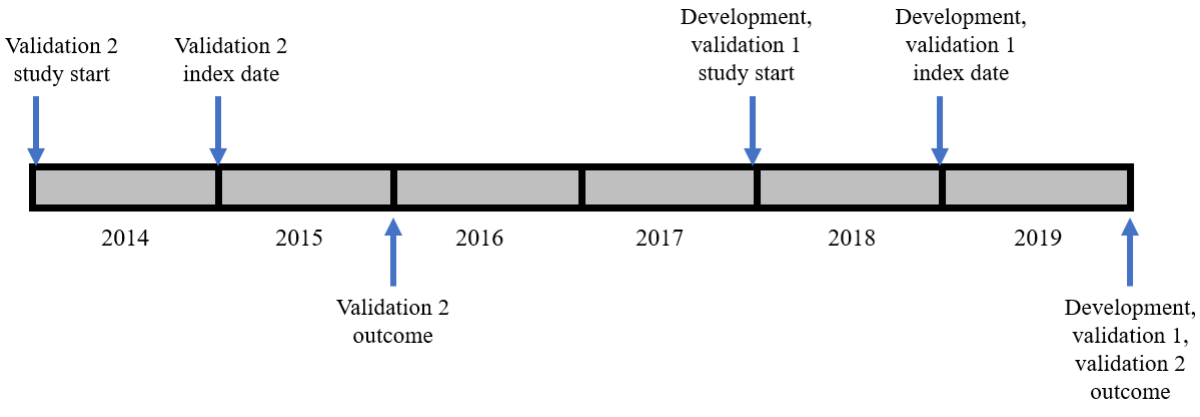
Study design for the development and validation of the GDPPR-version of the CMMS, involving 3 cohorts, with index dates set at 12 months after their respective study start dates, and synchronous and asynchronous outcomes at 1 year and 5 years (adapted from Tsang et al [21], which is published under Creative Commons Attribution 4.0 International License). CMMS: Cambridge Multimorbidity Score; GDPPR: General Practice Extraction Service Data for Pandemic Planning and Research.

We applied the same analytical approach and the same inclusion criteria as in our previous study, where we developed and validated a new CMMS for RSC using the more extensive list of SNOMED CT codes (we refer to this as the RSC-modified CMMS) [21]. We included people aged 16 years or older on the index date registered with a practice for 12 months or longer. Three separate data sets were sampled from the RSC as described previously (Figures S1 and S2 in Multimedia Appendix 1 [21]) and they are (1) derivation data set (n=500,000); (2) validation data set 1 (n=250,000) with the same study start and study end date as the derivation set (synchronous outcome); and (3) validation data set 2 (n=250,000) with 12-month outcome at a different time point to the derivation data set (asynchronous outcome), and 60-month outcome occurring at the same time point as the 12-month outcome of the derivation data set (synchronous outcome), as illustrated in Figure 1 [21]. These 3 data sets were generally comparable in terms of age, sex, number of conditions, and follow-up time.

### Curating and Selecting Individual CMMS Component Variables

**Figure 2:**
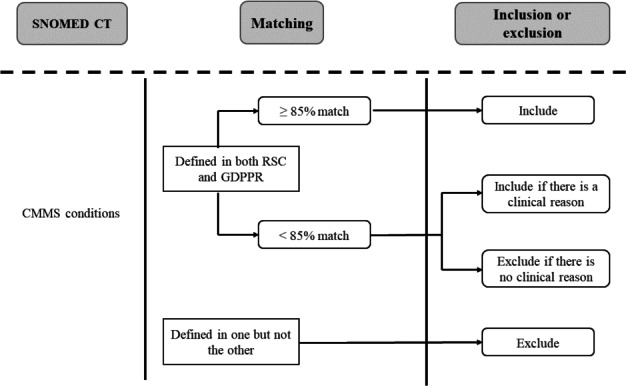
Selection of candidate CMMS conditions for the GDPPR-modified CMMS from the 37 conditions included in the original CMMS and inclusion of conditions based on prevalence defined by both the SNOMED CT lists available in the GDPPR and RSC, or knowledge that the condition is known to have high predictive value. CMMS: Cambridge Multimorbidity Score; GDPPR: General Practice Extraction Service Data for Pandemic Planning and Research; RSC: Research and Surveillance Centre; SNOMED CT: Systematized Nomenclature of Medicine clinical terms.

In selecting conditions for a new modified CMMS model, we first considered all 37 conditions that were included in the original CMMS development and validation and used the same definitions and prescriptions [17]. Following the approach we previously developed for the RSC-modified CMMS with SNOMED CT [21], we carefully curated the conditions within the limited set of SNOMED CT codes in the GDPPR. We performed a confirmatory study concerning SNOMED CT coverage in the GDPPR by carrying out a statistical and clinical matching of the conditions between the GDPPR and RSC (Figure 2). A matching percentage for each condition was defined as the prevalence ratio, that is, the prevalence in the RSC data set but included in the GDPPR set of SNOMED CT codes divided by the prevalence included in the RSC set of SNOMED CT codes. Therefore, a ratio less than 100% indicated a higher prevalence of that condition within the RSC data set using its set of RSC SNOMED CT codes, compared to the prevalence using the GDPPR SNOMED CT list on the same data set. We set an 85% threshold for inclusion in the development of the GDPPR-modified CMMS model unless there was a clinical reason to accept a lower threshold.

As the RSC provides a data set that is representative of England, we assumed that the actual prevalence of each condition in the RSC data set is similar to that in the GDPPR data set, and therefore, the RCS data set provided a suitable environment to develop the GDPPR-modified CMMS. We developed this GDPPR-modified CMMS in the RSC data set because it offered a complete set of SNOMED CT codes for each clinical concept, and we could replicate the reduced data set within GDPPR and then compare the case finding with each approach.

### Statistical Analyses

Using the previously described method [21], we used time-to-mortality Cox proportional hazards models based on the development data set. We first included the conditions as binary indicators with sex and age (in decades) and a quadratic age term as covariates. We then carried out a variable reduction process via backward elimination and using the Akaike information criteria as the stopping criterion [32]. The goal was to be parsimonious with the number of variables needed for implementation. This was carried out using the “fastbw” function from the rms R package. Model performance evaluation was based on discrimination and calibration. Discrimination was assessed by the pseudo *R*^2^, Somers D, and Harrell C [33]. Model calibration was evaluated by plotting a calibration curve and recalibration was carried out by resampling using cross-validation to correct for optimism or overfitting. This was implemented using the “calibrate” function in the rms R package. The model was developed, performance was evaluated on the derivation data set, and we then evaluated the performance of the models on the 2 validation data sets. All data preparation and analyses were conducted in R (version 4.1.0; R Core Team) [34], using the following R packages lme4 (version 1.1-27) [35], lubridate (version 1.7.10) [36,37], randomizr (version 0.20.0) [37], rms (version 6.2-0) [38], survival (version 3.2-11) [39,40], tableone (version 0.12.0) [41], and tidyverse (version 1.3.1) [42].

### Ethical Considerations

This development of the CMMS for use in GDPPR was performed as part of the RAVEN study which received ethical approval (Integrate Research Application Service number 300259) and was approved by the Health Research Authority’s Bromley Research Ethics Committee reference 21/HRA/1971, on October 8, 2021. NHS England hosts the national safe haven for patient data. The legal basis for this is Regulation 3 of the Health Service (Control of Patient Information Regulations) 2002 [43]. Pseudonymized data extracted from the practices are kept in a secured server at ORCHID, which is an NHS England policy-compliant trusted research environment (organization code EE133863-MSD-NDPCHS).

## Results

Of the 37 conditions in the original CMMS model, 22 were included in developing the GDPPR-modified CMMS (Table 1). This involved 5 conditions based on clinical judgement—alcohol problems, chronic liver disease and viral hepatitis, stroke and transient ischemic attack, thyroid disorders, and dementia—which were included for clinical reasons alone (Figure 2), as they were likely to have a good predictive value, notwithstanding their lower prevalence ratios. All but thyroid disorders remained in the model after variable reduction in the final 17-condition model (Table 1).

**Table 1:** Inclusion of the original CMMS^a^ set of 37 medical conditions in the 2 modified versions of the CMMS, for use in the RSC^b^ and for use in the GDPPR^c^, using the SNOMED CT^d^ list available for the 2 respective data sets, ordered by prevalence of these conditions in the RSC data set.

Condition	Patients in RSC data set as defined by RSC SNOMED CT codes (n=7,555,767), n	Prevalence ratio (%)	Final RSC-modified model^e^ (n=21)	Initial GDPPR- modified model (n=22)	Final GDPPR- modified model^e^ (n=17)
Diabetes	412,960	100.0	✓	✓	✓
Chronic kidney disease	260,270	100.0	✓	✓	✓
Chronis sinusitis	103,638	100.0		✓	
Bronchiectasis	34,050	99.9		✓	
Atrial fibrillation	198,949	99.7	✓	✓	✓
Asthma currently treated	542,001	99.0		✓	
Chronic obstructive pulmonary disease	147,191	99.0	✓	✓	✓
Schizophrenia or bipolar disease	48,975	97.9	✓	✓	✓
Epilepsy	54,964	95.9	✓	✓	✓
Parkinsonism	17,156	95.8	✓	✓	✓
Constipation	123,999	95.7	✓	✓	✓
Hypertension	1,280,958	94.7		✓	
Learning disability	44,100	93.4	✓	✓	✓
Heart failure	95,828	93.0	✓	✓	✓
Cancer in the last 5 years	168,577	91.1	✓	✓	✓
Peripheral vascular disease	38,946	87.3	✓	✓	✓
Coronary heart disease	322,338	84.7		✓	✓
Dementia	80,156	84.2	✓	✓	✓
Thyroid disorders	420,681	83.3		✓	
Stroke and transient ischemic attack	182,979	81.0		✓	✓
Migraine	33,719	68.4			
Connective tissue disorder or rheumatoid arthritis	153,350	51.8			
Chronic liver disease and viral hepatitis	52,265	49.3	✓	✓	✓
Painful conditions	810,458	43.6	✓		
Alcohol problems	190,439	33.0	✓	✓	✓
Psoriasis or eczema	63,556	26.5			
Inflammatory bowel disease	50,370	25.6			
Anxiety or depression	919,962	25.1	✓		
Disorders of prostate	201,559	25.0	✓		
Blindness and low vision	88,398	17.5			
Diverticular disease of intestine	229,536	17.2			
Anorexia	52,742	16.7			
Hearing loss	581,804	12.9			
Peptic ulcer disease	98,487	9.5			
Irritable bowel syndrome	407,880	0.0	✓		
Psychoactive substance misuse	92,944	0.0	✓		
Multiple sclerosis	15,717	0.0	✓		

^a^CMMS: Cambridge Multimorbidity Score.

^b^RSC: Research and Surveillance Centre.

^c^GDPPR: General Practice Extraction Service Data for Pandemic Planning and Research.

^d^SNOMED CT: Systematized Nomenclature of Medicine clinical terms.

^e^After variable reduction.

There were few differences in the development and validation data sets in terms of age, sex, number of conditions and follow-up time (Table 2).

The prevalence of the 22 conditions in the model derivation data set is presented in Table 3. These prevalences and their rankings were generally similar to those reported in the original CMMS study [17], and our previous study [21]. There was only 1 exception, coronary heart disease, which ranked lower (7163/500,000, 1.4%; original CMMS study—15,887/300,000, 4.8%; and previous RSC study—15,887/300,000, 5.3%).

The model performance of the 22-condition and 17-condition models was almost identical and similar to those in the original CMMS study and the RSC-modified CMMS (Tables 4 and 5).

**Table 2:** Descriptive statistics of 3 data sets sampled from the RSC^a^ for deriving and validating a modified version of the CMMS^b^ for use in the GDPPR^c^ data set, within the constraints of its limited set of SNOMED CT^d^ codes.

		Derivation (2019)		Validation 1 (2019)		Validation 2 (2015)
Male, n (%)		247,807 (49.6)		124,514 (49.8)		123,541 (49.4)
**Age at index date in years**						
	Mean (SD)	49.03 (19.29)	48.11 (19.09)		46.0 (19.34)	
	Range	16-95	16-95		16-95	
	65-84, n (%)	103,587 (22)	48,016 (0.21)		46,834 (0.20)	
	85 or older, n (%)	16,387 (4)	7,513 (0.03)		7,608 (0.03)	
**Number of conditions**						
	Mean (SD)	0.72 (1.19)	0.69 (1.17)		0.70 (1.0)	
	Range	0-11	0-11		0-11	
	0, n (%)	309,089 (62)	157,981 (63)		161,941 (65)	
	1, n (%)	101,547 (20)	49,463 (20)		47,530 (19)	
	2 or more, n (%)	89,364 (18)	42,556 (17)		40,529 (16)	
Number of deaths in follow-up, n		5104		2392		2408/11,948
Mean follow-up time^e^ (days), n		352.8		351.9		350.4/1538.0
Total person years^e,f^, n		482,885.5		240,859.6		239,859.2/1,052,567
Mortality rate (per 1000 person years)^e^, n		10.57		9.93		10.04/11.35

^a^RSC: Research and Surveillance Centre.

^b^CMMS: Cambridge Multimorbidity Score.

^c^GDPPR: General Practice Extraction Service Data for Pandemic Planning and Research.

^d^SNOMED CT: Systematized Nomenclature of Medicine clinical terms.

^e^1-year follow-up for validation 1 and 1- and 5-year follow-up for validation 2.

^f^Calculated as number of person-days divided by 365.25.

**Table 3:** Prevalence in individuals in the model derivation data set of the 22 candidate conditions in the GDPPR^a^-modified CMMS^b^ model before variable reduction and weights for the final set of 17 conditions after variable reduction, with conditions ordered by prevalence.

Condition	Value (n=500,000), n (%)	Weight
Hypertension	98,849 (19.8)	N/A^c^
Asthma currently treated	36,951 (7.4)	N/A
Diabetes	33,312 (6.7)	0.2623
Thyroid disorders	28,891 (5.8)	N/A
Chronic kidney disease	23,145 (4.6)	0.1286
Atrial fibrillation	15,041 (3.0)	0.2779
Cancer in the last 5 years	13,059 (2.6)	1.1876
Chronic obstructive pulmonary disease	12,734 (2.5)	0.6638
Alcohol problems	12,132 (2.4)	0.5670
Stroke and transient ischemic attack	12,118 (2.4)	0.2299
Constipation	8698 (1.7)	0.5889
Chronis sinusitis	8195 (1.6)	N/A
Coronary heart disease	21,897 (1.4)	0.1201
Heart failure	7163 (1.4)	0.5022
Dementia	5884 (1.2)	0.9815
Epilepsy	4114 (0.8)	0.6714
Schizophrenia or bipolar disorder	3819 (0.8)	0.5621
Learning disability	2857 (0.6)	1.0992
Peripheral vascular disease	2963 (0.6)	0.3519
Bronchiectasis	2563 (0.5)	N/A
Chronic liver disease and viral hepatitis	1890 (0.4)	1.0844
Parkinsonism	1409 (0.3)	0.5339

^a^GDPPR: General Practice Extraction Service Data for Pandemic Planning and Research.

^b^CMMS: Cambridge Multimorbidity Score.

^c^N/A: not applicable.

**Table 4:** Model discrimination for the final RSC^a^-modified and GDPPR^b^-modified versions of the CMMS^c^, after variable reduction, and compared with a full 37-condition model using RSC data and SNOMED CT^d^.

		37-condition model [21]		Final RSC-modified 21-condition model [21]		Final GDPPR-modified 17-condition model
Pseudo *R*^2^		0.153		0.153		0.140
Somers D		0.851		0.851		0.833
**Harrell C** **(SE)**						
	Derivation	0.925 (0.002)	0.926 (0.002)		0.916 (0.002)	
	Validation 1	0.920 (0.004)	0.921 (0.004)		0.922 (0.003)	
	Validation 2, 1-year follow-up	0.920 (0.003)	0.920 (0.003)		0.915 (0.003)	
	Validation 2, 5-year follow-up	0.907 (0.002)	0.907 (0.002)		0.902 (0.001)	

^a^RSC: Research and Surveillance Centre.

^b^GDPPR: General Practice Extraction Service Data for Pandemic Planning and Research.

^c^CMMS: Cambridge Multimorbidity Score.

^d^SNOMED CT: Systematized Nomenclature of Medicine clinical terms.

**Table 5:** Hazard ratios (95 CIs) of the predictors for the final RSC^a^-modified and GDPPR^b^-modified versions of the CMMS^c^, after variable reduction, and compared with a full 37-condition model using RSC data and SNOMED CT^d^.

	37-condition model HR^e^ (95% CI) [21]	RSC-modified 21-condition model HR (95% CI) [21]	GDPPR-modified 17-condition model HR (95% CI)
Age (10 years)	1.22 (1.02-1.47)	N/A^f^	1.02 (1.01-1.04)
[Age_(10 years)]^2^	1.05 (1.03-1.06)	1.06 (1.06-1.06)	1.00 (1.00-1.00)
Sex (Male)	1.33 (1.23-1.45)	1.34 (1.24-1.46)	1.14 (1.08-1.21)
Cancer in the last 5 years	3.31 (2.99-3.67)	3.33 (3.00-3.69)	3.28 (3.06-3.52)
Dementia	2.57 (2.33-2.84)	2.55 (2.32-2.82)	2.67 (2.47-2.88)
Alcohol problems	2.17 (1.84-2.55)	2.21 (1.88-2.60)	1.76 (1.53-2.03)
Multiple sclerosis	2.13 (1.32-3.44)	2.14 (1.33-3.46)	N/A
Chronic liver disease and viral hepatitis	1.98 (1.57-2.49)	1.99 (1.58-2.50)	2.96 (2.38-3.68)
Chronic obstructive pulmonary disease	1.96 (1.76-2.18)	2.02 (1.83-2.23)	1.94 (1.80-2.10)
Learning disability	1.88 (1.14-3.10)	1.89 (1.15-3.11)	3.00 (2.18-4.13)
Parkinsonism	1.71 (1.39-2.11)	1.73 (1.40-2.13)	1.71 (1.43-2.04)
Heart failure	1.66 (1.49-1.85)	1.66 (1.49-1.84)	1.65 (1.51-1.80)
Epilepsy	1.59 (1.25-2.02)	1.61 (1.27-2.04)	1.96 (1.62-2.37)
Schizophrenia or bipolar disorder	1.59 (1.22-2.06)	1.62 (1.25-2.10)	1.75 (1.42-2.17)
Psychoactive substance abuse	1.57 (1.20-2.04)	1.57 (1.20-2.04)	N/A
Painful condition	1.55 (1.42-1.68)	1.56 (1.44-1.69)	N/A
Constipation	1.47 (1.33-1.62)	1.47 (1.33-1.62)	1.80 (1.67-1.95)
Atrial fibrillation	1.39 (1.27-1.53)	1.40 (1.27-1.53)	1.32 (1.23-1.42)
Peripheral vascular disease	1.39 (1.07-1.81)	1.40 (1.08-1.82)	1.42 (1.24-1.63)
Anxiety or depression	1.38 (1.27-1.50)	1.38 (1.27-1.50)	N/A
Diabetes	1.31 (1.20-1.43)	1.34 (1.23-1.46)	1.30 (1.21-1.39)
Psoriasis or eczema	1.27 (1.03-1.57)	N/A	N/A
Chronic kidney disease	1.24 (1.14-1.35)	1.24 (1.14-1.35)	1.14 (1.06-1.21)
Anorexia or bulimia	1.22 (0.66-2.28)	N/A	N/A
Peptic ulcer	1.13 (0.98-1.30)	N/A	N/A
Bronchiectasis	1.11 (0.87-1.41)	N/A	N/A
Stroke and transient ischemic attack	1.11 (1.00-1.24)	N/A	1.26 (1.16-1.36)
Asthma currently treated	1.05 (0.93-1.18)	N/A	N/A
Hypertension	1.04 (0.96-1.13)	N/A	N/A
Thyroid disorder	1.03 (0.92-1.14)	N/A	N/A
Coronary heart disease	1.00 (0.91-1.09)	N/A	1.13 (1.05-1.21)
Chronic sinusitis	0.98 (1.57-2.49)	N/A	N/A
Rheumatoid arthritis	0.98 (0.85-1.12)	N/A	N/A
Blindness and low vision	0.96 (0.84-1.11)	N/A	N/A
Diverticular disease of intestine	0.92 (0.82-1.02)	N/A	N/A
Hearing loss	0.92 (0.95-1.00)	N/A	N/A
Disorder of the prostate	0.83 (0.74-0.93)	0.83 (0.74-0.93)	N/A
Irritable bowel syndrome	0.83 (0.71-0.95)	0.82 (0.71-0.94)	N/A
Inflammatory bowel disease	0.65 (0.43-0.97)	N/A	N/A
Migraine	0.59 (0.25-1.42)	N/A	N/A

^a^RSC: Research and Surveillance Centre.

^b^GDPPR: General Practice Extraction Service Data for Pandemic Planning and Research.

^c^CMMS: Cambridge Multimorbidity Score.

^d^SNOMED CT: Systematized Nomenclature of Medicine clinical terms.

^e^HR: hazard ratio.

^f^N/A: not applicable.

**Figure 3:**
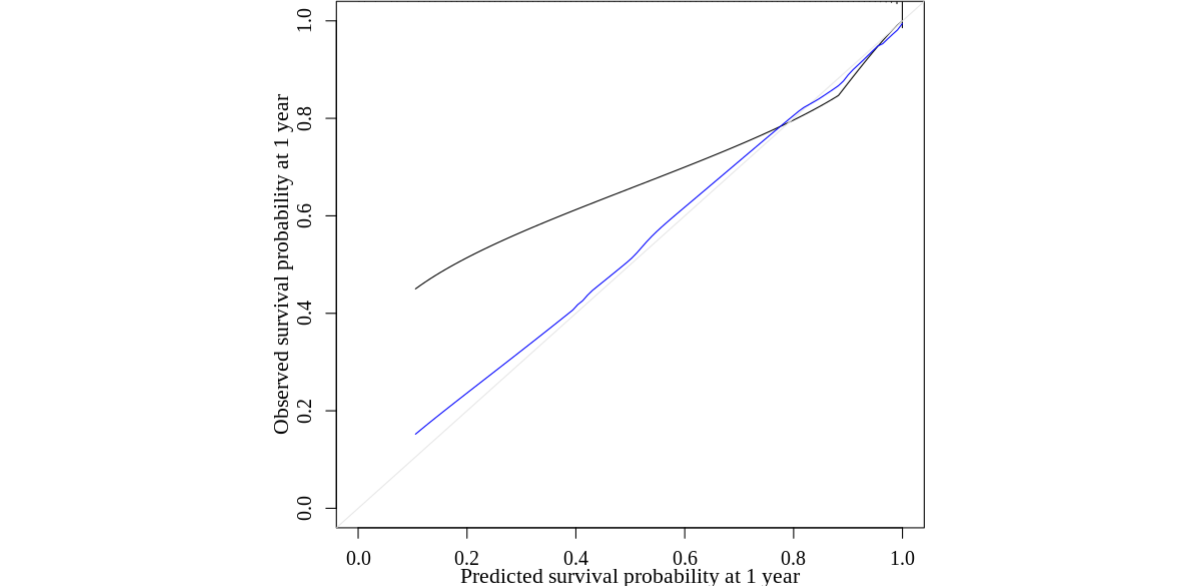
Calibration curve for the final 17 condition GDPPR-modified CMMS model. Black: observed; blue: optimism corrected; gray: ideal. Mean (SE) is 0.07 (0.9). Quantile is 0.009. CMMS: Cambridge Multimorbidity Score; GDPPR: General Practice Extraction Service Data for Pandemic Planning and Research.

For 1-year mortality, discrimination was high in both the derivation and validation data sets (Harrell C=0.92) and for 5-year mortality, it was slightly lower (Harrell C=0.90). The model calibration displayed underprediction at lower risks (<60%), and the calibration improved with the adjustment for optimism or overfitting (Figure 3).

## Discussion

### Principal Findings

In this study, we developed and validated a modified version of a single measure of multimorbidity, CMMS, for use within a national data set created during the pandemic from all existing primary care data collections, GDPPR, and using its limited set of SNOMED CT codes. The initial model included 22 conditions from the set of 37 in the original CMMS model and the reduced 17-condition model showed an identical performance in predicting mortality to the 22-condition model and a similar performance compared to the original 37-condition CMMS and the previous modification which was based on an extensive SNOMED CT data set.

### Interpretations and Implications

The GDPPR database remains available and is listed in the NHS Data Model and Dictionary [44]. It is now one of the data collections available through the NHS England Secure Data Environment [45], which was created as part of NHS England’s Data Saves Lives policy, following the Goldacre report [46].

This new single measure of multimorbidity will help us measure vaccine effectiveness using GDPPR, NHS England’s nationwide database. We will use this GDPPR-modified CMMS score in the RAVEN study to build on the existing evidence base [47,48]. Several observational studies have shown that the effectiveness of vaccination could be suboptimal in people with multimorbidity [49], and thus it is important to be able to explore and adjust for multimorbidity.

This tool may also be useful in a wider range of studies of people with multimorbidity, including vaccine and post-authorization safety studies. The risk of hospitalization, admission to intensive care unit beds, and mortality in people with multimorbidity are significantly higher than in the general population [50,51]. In an observational study of hospitalized patients in the United Kingdom with COVID-19, the crude mortality in people with multimorbidity, compared to single comorbidity, after adjusting for the demographic factors, was more than double (1492/3961, 37.7% vs 341/1971, 17.3%) [52]. It was estimated to reduce 63.5% (1905/3000) of deaths by prioritizing people with multimorbidity [53]. Therefore, people with multimorbidity were prioritized for vaccine rollout [54].

Our previous study showed that reducing the original CMMS variables from 37 to 21 did not compromise mortality predictability in people with multimorbidity [21]. In this study, we have demonstrated that the number of conditions can be reduced to 17 to match the data available in GDPPR and still can be a very good predictor of mortality.

### Comparison With Prior Work

We focused on mortality and this is the outcome most often reported in development studies of comorbidity indices [55]. There are many published comorbidity indices and they vary according to their time of development (and thus the number of modifications), derivation population, conditions (predictors), prediction horizon, outcome predicted, and data source [52]. Historically, the mortality indices have been designed for people in hospitals, using secondary care coding systems and they have provided predictions of in-hospital mortality and mortality between 6 months and 5 years [55]. The GDPPR-modified CMMS is the latest adaptation to the original CMMS [17] for predicting mortality in primary care. The predictions for the CMMS and its modified versions are for the same prediction horizons of 1 year and 5 years. The previous modification adapted the CMMS to conditions defined by the internationally recognized SNOMED CT coding system [21], as the original CMMS was based on a population in the United States and conditions defined by the Read clinical terminology, which is no longer used in England. Both modifications of the CMMS have been developed on English populations with a lower minimum age compared to that for the original version (16 years as opposed to 21 years).

This GDPPR-modified version produces a multimorbidity index for mortality based on conditions defined by the limited SNOMED CT list of the GDPPR. The RSC provided the development data set for both modifications of the CMMS. The RCS has a complete set of SNOMED CT codes. Its data set includes people registered in a fraction of English general practices, and the assumption of this study is that the underlying prevalence of each CMMS condition in people in the RSC data set is the same as those in the larger GDPPR data set. While the RSC is recruited to be nationally representative, there may inevitably be differences [24].

The GDPPR includes the English primary care data used by the British Heart Foundation’s Data Science Centre for their COVID-19 and cardiovascular diseases Consortium’s work. Our version of CMMS could be deployed by this and other groups using GDPPR or underlying primary care (General Practice Extraction Service) data [56].

### Strengths

The main strength of this study is that it built on our expertise in developing a version of CMMS that could be applied to routine clinical data recorded using SNOMED CT. This terminology is used internationally [21]. We overcame the limitations of the relatively limited number of SNOMED clinical terms in GDPPR and demonstrated that a 17-condition CMMS could run in the GDDPR data set of 54 million individuals.

### Limitations

There are several limitations of this study. We only predicted the mortality risk and did not predict the hospitalization or intensive care unit admission risk. The conditions were a subset of those included in the original CMMS study, which arose from a review of multimorbidity literature at the time of its development. The nature of disease and treatments, as to population characteristics, will change over time. Hence, the new CMMS versions will need to be updated regularly, and this may involve adding conditions that were not included in the original CMMS. Although we split our initial data set randomly into development and validation data sets, we have performed simple temporal external validation [57]. A more robust form of external validation would involve investigating the generalizability to other countries (geographical validation) or other settings (domain validation), but neither is relevant in this case as we are considering a national data set, and we have developed the new CMMS using what we assume to be a representative sample of the adult English population. The generalizability could be tested further on other primary care data such as Clinical Practice Research Datalink [18], which provides a database of anonymized health records for another sample of English general practitioner practices.

### Conclusions

This latest modification of the CMMS provides a new validated single multimorbidity measure, which was generated through a combination of unique access to data and expertise in validation. The RSC provided nationally representative and comprehensive primary care data. The study team had experience in developing a validated CMMS version to use within SNOMED CT. This combination meant that it was possible to develop and validate a new version of CMMS for use in the national English data set, the GDPPR. Our previous study showed that reducing the original CMMS variables from 37 to 21 did not compromise mortality predictability in people with multimorbidity [21]. In this study, we have demonstrated that the number of conditions can be reduced to 17 to match the data available in GDPPR and still can be a very good predictor of mortality. Therefore, researchers using this national database, or looking for a further reduced CMMS measure, can use this 17-component single measure of comorbidity.

The approach used in this study could also be applied in other contexts. Our approach has been to replicate a validated multimorbidity measure in a smaller, but complete and high data quality sentinel network database, the RSC. Within the RSC we could ensure the model performs as well as the one run on complete data [21]. Additionally, developing and validating this reduced CMMS model in the RSC required less processing time. This may make this reduced version more attractive to other users, should processing time be at a premium.

## Appendix

Multimedia Appendix 1 Flowcharts showing the selection of practices and individuals for inclusion in the analysis (adapted from Tsang et al [21], which is published under Creative Commons Attribution 4.0 International License).

## Abbreviations

CCI: Charlson Comorbidity Index
CMMS: Cambridge Multimorbidity Score
GDPPR: General Practice Extraction Service Data for Pandemic Planning and Research
LTC: long-term condition
NHS: National Health Service
ORCHID: Oxford Royal College of General Practitioners Digital Informatics Hub
RAVEN: real-world effectiveness of the Oxford-AstraZeneca COVID-19 vaccine in England
RCGP: Royal College of General Practitioners
RSC: Research and Surveillance Centre
SNOMED CT: Systematized Nomenclature of Medicine clinical terms

## Data Availability

The data sets generated and analyzed during this study are not publicly available as they have to be analyzed within a secure network, after appropriate training and subject to ethical approval, and this can be arranged by the corresponding author on reasonable request.
