# Supplementary material for: Creating a Modified Version of the Cambridge Multimorbidity Score to Predict Mortality in People Older Than 16 Years: Model Development and Validation
Source: J Med Internet Res. 2024 Aug 26;26:e56042. doi: 10.2196/56042 (PMC11384182; doi:10.2196/56042)
Supplement: Multimedia Appendix 1 [file jmir_v26i1e56042_app1.docx]

Supplementary files

**Figure S1. Flow diagram for selection of practices for the development and validation of the GDPPR-modified version of the CMMS**


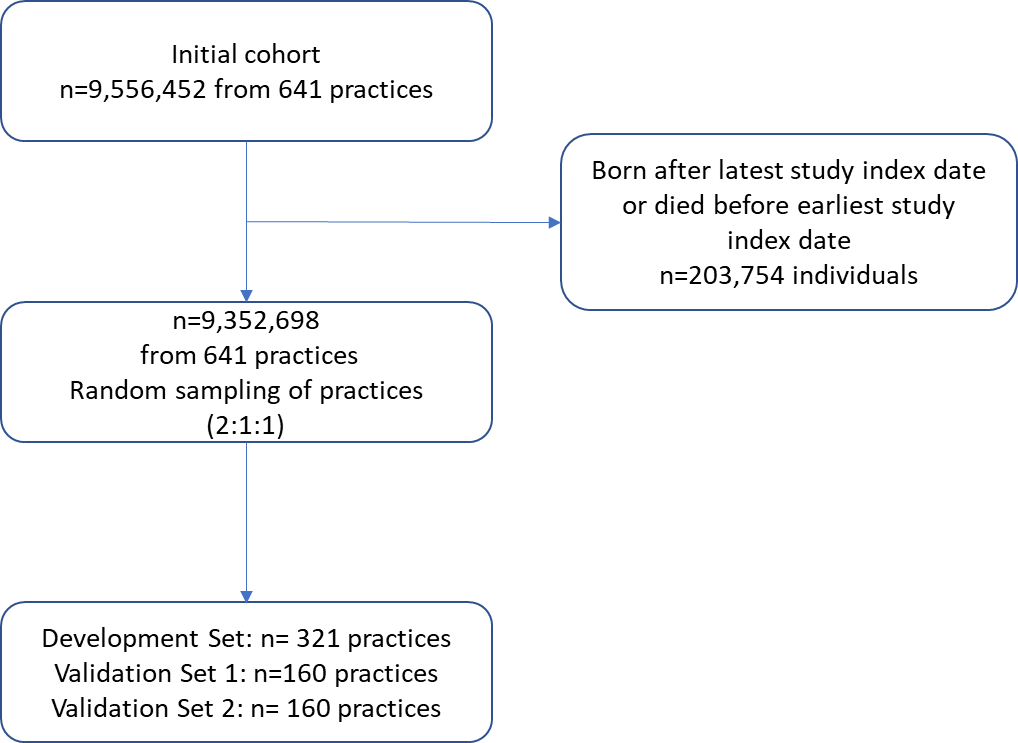


Adapted with permission – Tsang et al., 2022 [21]

**Figure S2. Flow diagram for selection of individuals for the development and validation of the GDPPR-modified version of the CMMS**


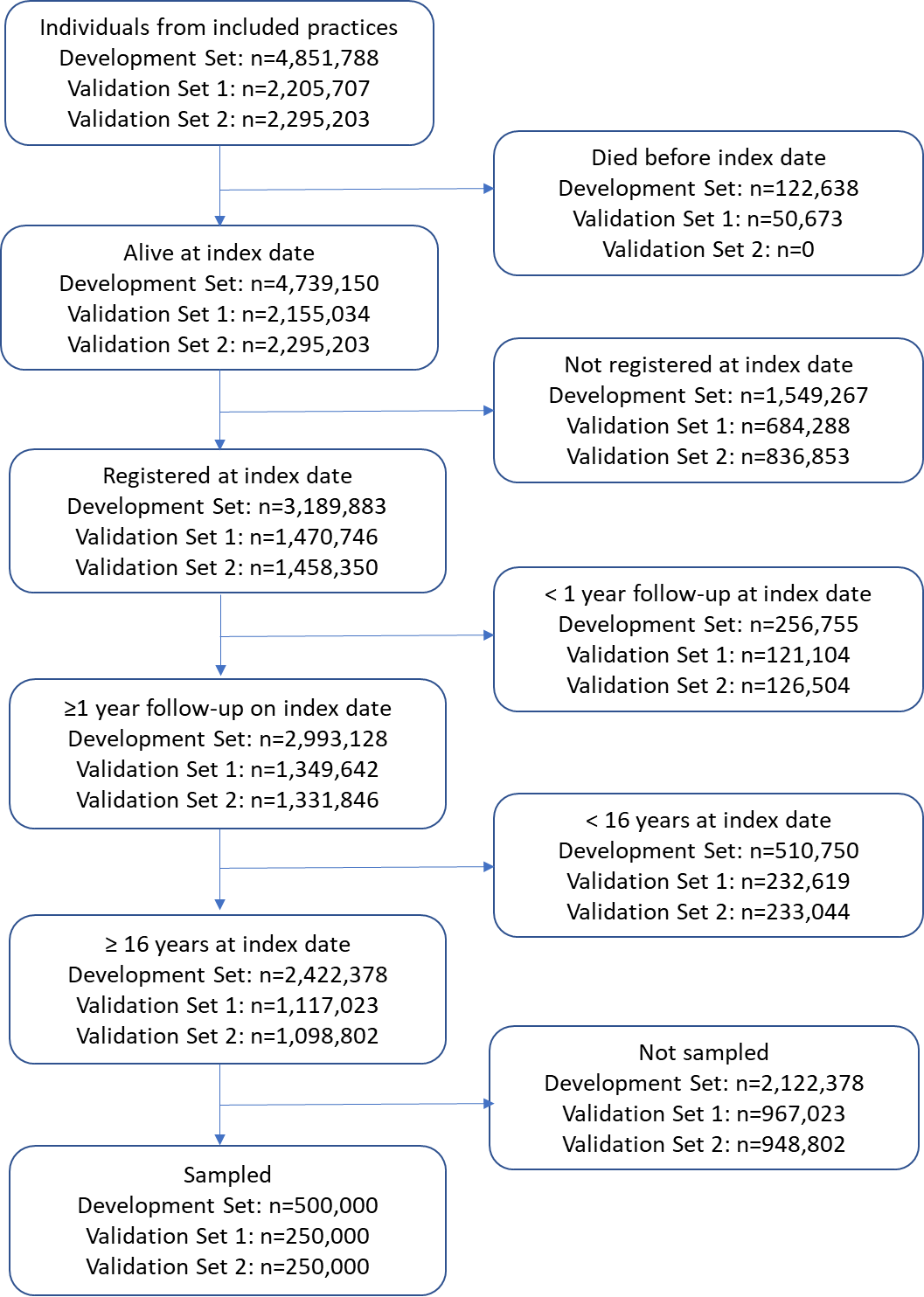


Adapted with permission – Tsang et al., 2022 [21]
